# Stability comparison of two different dentoalveolar expansion treatment protocols

**DOI:** 10.1590/2177-6709.22.5.075-082.oar

**Published:** 2017

**Authors:** Ezgi Atik, Tülin Taner

**Affiliations:** 1 Assistant Professor in Orthodontics, School of Dentistry, University of Hacettepe (Ankara, Turkey).; 2 Professor in Orthodontics, School of Dentistry, University of Hacettepe (Ankara, Turkey).

**Keywords:** Conventional brackets, Self-ligating brackets, Stability

## Abstract

**Objective:**

The aim of this study was to compare the longitudinal stability of the conventional straight-wire system after the use of a quad-helix appliance with Damon self-ligating system in patients with Class I malocclusion.

**Methods:**

27 adolescent patients were evaluated at three different periods: pre-treatment (T_1_), post-treatment (T_2_) and three years post-treatment (T_3_). Group 1 included 12 patients (with a mean age of 14.65 year) treated with Damon 3MX bracket system; and Group 2 included 15 patients (with a mean age of 14.8 year) who underwent orthodontic treatment with Roth prescribed brackets after expansion with Quad-Helix appliance. Relapse was evaluated with dental cast examination and cephalometric radiograph tracings. Statistical analysis was performed with IBM-SPSS for Windows software, version 21 (SPSS Inc., Chicago, IL). A *p*-value smaller than 0.05 was considered statistically significant.

**Results:**

There were significant increases in all transverse dental and postero-anterior measurements (except for UL6-ML mm in Group 1) with active treatment. There was some significant relapse in the long-term in inter-canine width in both groups and in the inter-first premolar width in Group 2 (*p*< 0.05). Significant decrease in all frontal measurements from T_2_ to T_3_ was seen for both groups. Upper and lower incisors significantly proclined in T_1_-T_2_ (*p*<* *0.05), however no relapse was found for both groups. When two systems were compared, there was no significant difference for the long-term follow-up period.

**Conclusion:**

Conventional (quad-helix appliance with conventional brackets) and Damon systems were found similar with regard to the long-term incisor positions and transverse dimension changes of maxillary arch.

## RESUMO

**Objetivo:**

comparar a estabilidade longitudinal após o tratamento de pacientes com má oclusão de Classe I usando o sistema *Straight-wire* convencional - depois da expansão com aparelho quadri-hélice - e o sistema autoligável Damon.

**Métodos:**

27 pacientes adolescentes foram avaliados em três períodos distintos: pré-tratamento (T_1_), pós-tratamento imediato (T_2_) e três anos pós-tratamento (T_3_). O Grupo 1 incluiu 12 pacientes (com idade média de 14,65 anos) tratados com o sistema de braquetes Damon 3MX e o Grupo 2 incluiu 15 pacientes (com idade média de 14,8 anos), submetidos a tratamento ortodôntico com braquetes prescrição Roth após expansão com aparelho quadri-hélice. A recidiva foi avaliada por meio de exame dos modelos de estudo e traçados cefalométricos. A análise estatística foi realizada com o *software* IBM-SPSS para Windows, versão 21 (SPSS Inc., Chicago, IL). Valores de *p*< 0,05 foram considerados estatisticamente significativos.

**Resultados:**

após o tratamento ativo, ocorreu aumento significativo em todas as medidas transversais dentárias e posteroanteriores (exceto para a UL6-ML mm, no Grupo 1). Em longo prazo, ocorreu recidiva significativa (*p*< 0,05) na distância intercaninos em ambos os grupos, e na distância interprimeiros pré-molares no Grupo 2. De T_2_ para T_3_, observou-se diminuição significativa em todas as medidas frontais, para ambos os grupos. De T_1_ para T_2_, os incisivos superiores e inferiores sofreram vestibularização significativa (*p*< 0,05); porém, nenhuma recidiva ocorreu em qualquer um dos dois grupos. Ao se comparar os dois sistemas, não foi encontrada qualquer diferença significativa no período de acompanhamento em longo prazo.

**Conclusão:**

o sistema convencional (aparelho quadri-hélice e braquetes convencionais) e o sistema Damon apresentaram desempenho semelhante, em longo prazo, em termos das posições dos incisivos e das mudanças ocorridas na dimensão transversal da arcada superior.

## INTRODUCTION

One of the important aspects of orthodontic treatment is to maintain arch form and prevent the possibility of relapse. However, increasing the arch perimeter in non-extraction orthodontic treatment results in both transverse expansion of the arches and proclination of the incisors.^1^ ^,^ ^2^ On the other hand, it is known that arch dimensional changes most probably influence prolonged stability. Both widening the inter-canine dimension width and tipping incisors labially results in unstable post-treatment results.^3^ ^,^ ^4^

Posterior expansion in the maxillary and mandibular arches is one of the comparison issues of self-ligating and conventional brackets. Transverse expansion with self-ligating systems is explained by low friction between the brackets and the archwires.^5^ In the literature, some studies have indicated greater arch width increases with self-ligating brackets,^6^ ^-^ ^8^ while other studies have shown no differences between self-ligating and conventional appliances.^9^ ^-^ ^11^ It has been purported that the lower force produced by self-ligating brackets might lead to more stable treatment results.^12^ It has also been claimed that passive self-ligating brackets can introduce stable arch dimensional changes.^13^ However, few studies^14^ ^,^ ^15^ in the literature have evaluated the stability of treatment results associated with self-ligating bracket systems.

Since there is a lack of studies comparing the long-term stability of conventional and self-ligating systems, the present study aimed to comparatively evaluate the long-term post-treatment effects of self-ligating and conventional systems on the transverse dimensions of maxillary arches, and identify the dentoalveolar cephalometric changes. The null hypothesis assumed that there was not significant differences regarding the long-term stability between both systems. 

## MATERIAL AND METHODS

The subjects in this study were derived from a sample of 33 patients who had been previously treated in Hacettepe University Department of Orthodontics. The treatment results were originally presented in other article.^9^ The following inclusion criteria were used: patients were 13-17 years of age at the start of treatment; they had Class I malocclusion (ANB angle between 2° and 4°); moderate maxillary and mandibular crowding (between 3 mm and 6 mm); maxillary constriction caused by dental transverse discrepancy, characterized by palatal tipping of the upper premolar and/or molar teeth; had undergone the non-extraction treatment protocol using the same archwire sequence with self-ligating brackets (Damon 3MX, Ormco/A Company, San Diego, CA, USA) or quad-helix expansion followed by conventional brackets (Forestadent, Pforzheim, Germany); had not undergone any adjunctive method, such as stripping, power chain, or intermaxillary elastics so as not to constrict the maxillary arch; and had undergone the same retention protocol in both the upper and lower dental arch for approximately one year. All patients had available records from before treatment (T_1_), after treatment (T_2_), and three years after treatment (T_3_). In this retrospective clinical study, no sample size calculation was done because all the available records were included. The power analysis was done and the power of the effect of different treatment protocols according to the obtained results and hypothesis was found to be 99.47%. 

Ethical approval was obtained from the University of Hacettepe Research Committee (No. GO 16/573-13). A total of 33 patients were recalled to the Orthodontic Department of Hacettepe University, for a follow-up investigation three years post-treatment. Previously, the patients were randomly allocated to one of the two treatment systems. Six patients did not return for long-term post-treatment records. Therefore, the final number of follow-up patients was 27; 12 subjects were assigned to the Damon bracket group (Group 1) and 15 subjects were assigned to the conventional bracket group (Group 2), as shown in Table 1. In the present study the pre-treatment and post-treatment variables were not obtained from the aforementioned previous study^9^, being re-evaluated, since the sample size was smaller in this study.

**Table 1:** Demographic and clinical characteristics of the sample.

Variables	Group 1 (Damon)	Group 2 (Conventional)	p-value
Number of subjects	12	15	
Age (year)	14.65 (13.10-16.70)	14.80 (12.10-15.90)	0.905^a^
Mand. crowding (mm)	3.75±0.94	3.31±0.74	0.256^a^
Max. crowding (mm)	4.24±1.14	3.66±0.90	0.236^a^

a: Mann-Whitney test. Values are presented as mean ± standard deviation (SD), median (min-max) or p-value.

The Damon bracket group (12 female patients with a mean age of 14.65 years and an age range of 13.1-16.7 years) was previously bonded with a 0.022-in Damon 3MX appliance system (Ormco/A Company, San Diego, CA, USA). Unlike the conventional bracket group, in the Damon bracket group the application of the expansion appliance was not performed before the bonding procedure. The following archwires were used, sequentially, for leveling and aligning: 0.014, 0.018-in Damon copper-nickel-titanium (CuNiTi); 0.014×0.025, 0.017×0.025-in Damon CuNiTi, followed by 0.017×0.025-in and 0.019×0.025-in stainless steel (SS) archwires.

The conventional bracket group (15 female patients with a mean age of 14.8 years and an age range of 12.1-15.9 years) was previously bonded with the 0.022-in Roth prescription bracket (Forestadent, Pforzheim, Germany). At the beginning of the treatment, the maxillary arch was expanded using a quad-helix appliance until the lingual cusps of the maxillary first molars were in contact with the buccal cusps of the mandibular first molars. After the desired expansion of the maxillary arch was achieved, the quad-helix appliance was removed and the transpalatal arch, with two arms behind the upper right and left premolar teeth, was put in place for retention until the SS archwires were applied to the maxillary arch. The following sequence of archwires was used for leveling and aligning: 0.014, 0.018-in CuNiTi; 0.014 × 0.025, 0.017 × 0.025-in CuNiTi, followed by 0.017 × 0.025 and 0.019 × 0.025-in SS. In the Damon bracket group, the Damon CuNiTi archwires were in uniform archwire form, and they were not coordinated to the original dental arch form. In the conventional bracket group, standard CuNiTi archwires were used. When these two types of archwires were compared, they displayed the same shape in the front region; however, the Damon CuNiTi archwires were wider in the region distal to the canines. In the Damon bracket group, Damon SS archwires were used; in the conventional bracket group, medium arch form SS archwires were used. The Damon SS archwires were broader than the standard SS archwires used in the conventional bracket group.

When the treatment was completed, upper and lower Hawley retainers were applied to all patients, for the retention protocol. The devices were made with Orthocryl^®^ (Dentaurum, Ispringen, Germany). The patients were instructed to wear the Hawley retainers full time for six months, except when eating and brushing their teeth and thereafter 6 months for every night. The total retention period was one year. The patients were evaluated at three-month intervals until the completion of the retention period (one year) to determine their motivation, address hygiene issues, and assess breakage.

**Figure 1:**
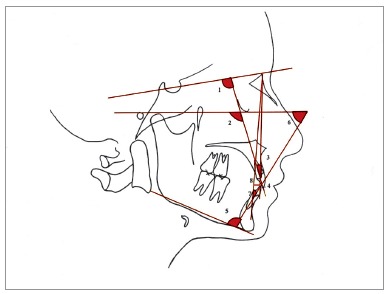
Lateral cephalometric dental angular and linear measurements: 1) U1-SN (degrees); 2) U1-FH (degrees); 3) U1-NA (degrees); 4) U1-NA (mm); 5) IMPA (degrees); 6) FMIA (degrees); 7) L1-NB (degrees); 8) L1-NB (mm).

**Figure 2:**
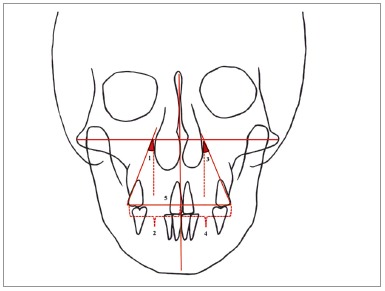
Posteroanterior cephalometric measurements: 1) UR6-ML (degrees); 2) UR6-ML (mm); 3) UL6-ML (degrees); 4) UL6-ML (mm); 5) UR6-UL6 (mm).

**Figure 3:**
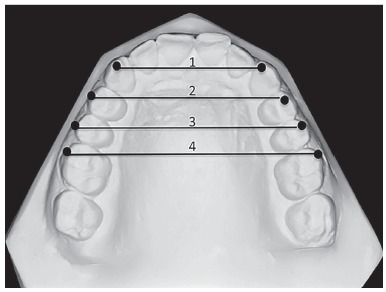
1) Inter-canine width (3-3), which is the distance between the right and left maxillary canine cusp tips. 2) Inter-first premolar width (4-4), which is the distance between the buccal cusp tips of the right and left maxillary first premolars. 3) Inter-second premolar width (5-5), which is the distance between the buccal cusp tips of the right and left maxillary second premolars. 4) Inter-molar width (6-6), which is the distance between the mesiobuccal cusp tips of the right and left maxillary first molars.

All records, including postero-anterior and lateral cephalometric radiographs - with the use of the same cephalostat (Promax; Planmeca, Helsinki, Finland) - were obtained from all patients. Dental casts prepared from alginate impressions were also obtained from all patients before treatment (T_1_) and immediately after treatment (T_2_). When the patients were recalled three years after the completion of the treatment, the same records were taken by the same operator, for post-retention evaluation (T_3_). Eight lateral cephalometric measurements (Fig 1), five frontal cephalometric measurements (Fig 2), and four dental cast measurements (Fig 3) were obtained and recorded. Digital tracing of the cephalometric radiographs using Quick Ceph Studio software (Quick Ceph System, San Diego, CA, USA) and dental model measurements were performed by the same investigator. The transverse dimensions were recorded with digital calipers (150 mm ISO 9001 electronic caliper; Tesa Technology, Renens, Switzerland), as shown in Figure 3. 

### Statistical analysis

Descriptive and analytical statistical analyses were performed with IBM-SPSS for Windows software, version 21 (SPSS Inc., Chicago, IL, USA). The Shapiro-Wilk test was used to determine if the continuous data were normally distributed. Data were shown as mean ± standard deviation or median (min-max), where applicable. Degrees of reliability were calculated by intra-class correlation coefficients and 95% confidence interval for each clinical parameter: the degree of concordance observed was classified as satisfactory and excellent, respectively.

Mann-Whitney test was used to compare the demographic variables among the groups. Two-way repeated ANOVA measure test was used to compare the differences among the groups. The non-parametric Friedman test was used to evaluate the statistical significance of the mean differences between the pre-treatment, post-treatment, and post-retention measurements within the groups. A p-value smaller than 0.05 was considered to be statistically significant.

## RESULTS 

All the dental arch width measurements were found to have significantly increased from the T_1_ period to T_2_ period in both groups (*p*< 0.05). From T_2_ to T_3_, there was some significant relapse in inter-canine width in both groups (*p*< 0.05) and in the inter-first premolar width in Group 2, the conventional bracket group (*p*= 0,019) (Table 2). 

**Table 2:** Dental Model Meausurements of the Groups at T1 (pre-treatment) ,T2 (post-treatment) and T3 (3 years post-treatment) Periods

Variables	T_1_	T_2_	T_3_	T_2_-T_1_	T_3_-T_2_	T_3_-T_1_	
	mean±SD	mean±SD	mean±SD	p^a^	p^a^	p^a^	p^b^
3-3							0,647
Group 1 (Damon)	33.83±3.54	35.99±2.41	34.81±2.34	0.001*	0.007*	1.000	
Group 2 (Conventional)	32.861±3.15	34.94±1.93	34.08±1.20	0.0001*	0.019*	0.301	
4-4							0.438
Group 1 (Damon)	38.16±2.95	43.58±2.13	42.35±1.98	0.0001*	0.662	0.007*	
Group 2 (Conventional)	38.09±2.32	44.03±2.06	42.24±2.20	0.0001*	0.019*	0.019*	
5-5							0.462
Group 1 (Damon)	42.82±3.49	48.28±2.76	47.16±2.30	0.0001*	0.662	0.007*	
Group 2 (Conventional)	43.79±3.38	49.12±2.26	47.47±2.49	0.0001*	0.053	0.010*	
6-6							0.976
Group 1 (Damon)	48.85±3.73	52.70±3.34	51.75±2.89	0.0001*	0.662	0.007*	
Group 2 (Conventional)	49.90±3.90	53.77±2.91	52.73±3.18	0.0001*	0.134	0.006*	

*Statistically significant (*p*< 0,05).

a: Friedman test, comparison of pre-treatment, post-treatment and 3 years post-treatment measurements within the groups.

b: Two-way repeated ANOVA measure test, comparison of groups.

Statistically significant increases were found in the postero-anterior measurements (except for UL6-ML mm in the Damon bracket group, Group 1) in the T_1_-T_2_ period for both groups. Moreover, there was a significant decrease (*p*< 0.05) in all the frontal measurements in the T_2_-T_3_ period for both groups (Table 3).

**Table 3:** Postero-anterior cephalometric measurements of the groups at T_1_ (pre-treatment), T_2_ (post-treatment) and T_3_ (3 years post-treatment) periods.

Variables	T_1_	T_2_	T_3_	T_2_-T_1_	T_3_-T_2_	T_3_-T_1_	
	mean±SD/ median (min-max)	mean±SD/ median (min-max)	mean±SD	p^a^	p^a^	p^a^	p^b^
UR6-ML (mm)							0,252
Group 1	27.98±1.86	29.86±2.18	26.53±1.41	0.013*	0.0001*	0.922	
Group 2	30.13±1.79	31.23±2.22	27.98±2.73	0.019*	0.000*	0.301	
UR6-ML (degrees)							0.164
Group 1	24.47±2.97	30.01±2.67	24.26±3.03	0.013*	0.0001*	0.922	
Group 2	26.96 (16.18-30.64)	29.61 (20.57-31.77)	24.52±3.49	0.003*	0.0001*	1.000	
UL6-ML (mm)							0.501
Group 1	28.57±1.22	29.61±1.23	26.33±0.99	0.074	0.0001*	0.074	
Group 2	29.31±2.10	30.78±1.74	27.53±2.53	0.010*	0.0001*	0.604	
UL6-ML (degrees)							0.055
Group 1	27.62±3.64	31.68±3.03	26.20±4.12	0.002*	0.0001*	1.000	
Group 2	26.79±3.82	29.44±2.97	26.73±2.32	0.002*	0.006*	1.000	
UR6-UL6 (mm)							0.831
Group 1	56.97±2.64	59.34±2.80	53.71±2.16	0.043*	0.0001*	0.199	
Group 2	59.59±3.45	61.73±3.56	56.62±5.40	0.002*	0.0001*	1.000	

*Statistically significant (*p*< 0,05).

a: Friedman test, comparison of pre-treatment, post-treatment and 3 years post-treatment measurements within the groups.

b: Two-way repeated ANOVA measure test, comparison of groups.

In the T_1_-T_2_ period, U1-SN (degrees), U1-FH (degrees), FMIA (degrees), L1-NB (degrees), and L1-NB (mm) significantly changed in Group 1; and U1-NA (degrees), IMPA (degrees), FMIA (degrees), L1-NB (degrees), and L1-NB (mm) significantly changed in Group 2. From T_2_ to T_3_, no significant relapse was found in the lateral cephalometric measurements for both groups (Table 4). 

**Table 4:** Lateral cephalometric measurements of the groups at T_1_ (pre-treatment), T_2_ (post-treatment) and T_3_ (3 years post-treatment) periods.

Variables	T_1_	T_2_	T_3_	T_2_-T_1_	T_3_-T_2_	T_3_-T_1_	
	mean±SD	mean±SD	mean±SD	p^a^	p^a^	p^a^	p^b^
U1-SN (degrees)							0,173
Group 1	102.47±8.41	107.07±7.35	106.92±5.70	0.007*	1.000	0.043*	
Group 2	102.00±7.17	103.48±5.73	103.40±7.50	0.448	1.000	0.08	
U1-FH (degrees)							0.342
Group 1	112.76±7.20	116.99±6.09	116.80±5.48	0.024*	1.000	0.074	
Group 2	111.86±6.70	113.09±5.54	114.12±6.37	0.432	0.706	0.024*	
U1-NA (degrees)							0.386
Group 1	22.08±7.80	27.00±5.30	27.42±6.10	0.074	1	0.124	
Group 2	22.86±6.65	25.16±5.16	25.16±7.29	0.018*	0.523	0.083	
U1-NA (mm)							0.45
Group 1	4.64±2.59	6.06±1.61	6.15±1.46	0.074	1.000	0.024*	
Group 2	5.13±2.18	5.86±2.45	5.64±2.18	0.053	1	0.249	
IMPA (degrees)							0.867
Group 1	95.70±3.58	101.41±6.24	100.50±5.30	0.074	0.922	0.003*	
Group 2	92.95±6.43	97.56±7.70	97.35±7.92	0.019*	1.000	0.019*	
FMIA (degrees)							0.764
Group 1	61.27±6.97	54.05±6.78	55.91±8.31	0.024*	1	0.013*	
Group 2	62.47±6.24	56.56±6.71	57.66±7.66	0.002*	1	0.002*	
L1-NB (degrees)							0.702
Group 1	26.08±4.72	32.55±5.14	30.90±6.11	0.002*	1.000	0.003*	
Group 2	23.84±5.90	28.77±6.58	27.98±7.18	0.002*	1.000	0.006*	
L1-NB (mm)							0.128
Group 1	5.40±2.65	7.77±2.58	7.41±3.01	0.001*	1.000	0.007*	
Group 2	5.17±2.22	6.76±2.37	6.17±2.50	0.000*	0.134	0.067	

*Statistically significant (*p*< 0,05).

a: Friedman test, comparison of pre-treatment, post-treatment and 3 years post-treatment measurements within the groups.

b: Two-way repeated ANOVA measure test, comparison of groups.

The inter-group comparison results showed that there was no statistically significant difference between the groups for all measurements (Table 2, 3 and 4).

## DISCUSSION

In orthodontic treatment, it is difficult to sustain the patient’s dental arches in the position attained by active treatment. Several reasons might cause the tendency toward relapse, such as inter-canine width, mandibular growth rotation, third molar eruption, and different treatment patterns.^16^ ^-^ ^19^

While evaluating the long-term stability of an orthodontic treatment, the pattern and magnitude of the dentoalveolar arch dimensional changes must be taken into consideration. The increase in the inter-canine arch width and proclination of the incisors are the main causes of unstable results.^3^ ^,^ ^20^ Therefore, it is important to maintain the arch form during orthodontic treatment, and doing so is highly recommended. There seems to be little basis for the claim that self-ligating brackets induce stable dental arch expansion.^13^

The effects of self-ligating brackets on long-term stability are largely unknown due to the lack of sufficient long-term follow-up studies. For this reason, the current study evaluated the three-year post-treatment stability of self-ligating and conventional treatment systems on patients that had been previously treated. All the patients included in the current study had a dentally-constricted maxillary arch with Class I malocclusion. Therefore, in the conventional bracket group, the quad-helix expansion appliance was used for transverse expansion of the maxillary arch before the alignment of the teeth with straight-wire appliances, including conventional brackets. However, in the Damon bracket group, due to the expansion feature of the Damon CuNiTi archwires,^21^ the expansion appliance was not used before the leveling and alignment stages. 

 In both treatment systems, the arch widths were significantly larger post-treatment. Although an expansion appliance was not used in the Damon bracket group (Group 1), the increase in the arch widths in that group can be attributed to the larger CuNiTi and SS archwires in the region distal to the canines. In the three-year post-treatment period (from T_2_ to T_3_), significant relapse in the inter-canine width was observed in both groups, and relapse was observed in the inter-first premolar width in the conventional bracket group (Group 2) (Table 3). The inter-canine width relapse amounts were 1.18 mm and 0.86 mm for Group 1 and Group 2, respectively; the inter-first premolar width decrease was 1.79 mm for Group 2. Several studies have shown an inter-canine and inter-molar width decrease during the post-retention period when it had been expanded during active treatment.^22^ ^-^ ^24^ In the present study, the significant relapse in the inter-canine width in both groups after the post-treatment phase may be due to the constriction of the expanded inter-canine dimension.^25^ This relapse could also be explained by the disuse of the Hawley appliances after one year, since the use of removable appliances was under the control of the patients. In addition, when the two groups were compared, the arch width decrease in the self-ligating brackets group (Group 1) did not differ from the arch width decrease in the conventional bracket group (Group 2) after the post-treatment period. Yu et al^14^ compared the long-term stability of treatment with self-ligating brackets and conventional brackets with a mean follow-up period of 7.68 years. Different from the present study, these authors^14^ found a greater increase in the inter-molar width with self-ligating brackets than with conventional appliances. This different result can be attributed to different treatment modalities, such as the expansion appliance used with the conventional brackets in the present study. Similar to the results of the present study, they^14^ did not find a significant difference in the inter-canine and inter-molar width change between the bracket systems over the long-term follow-up period. This result is concurrent with our study, and indicates that the bracket type do not affect the stability associated with dental arch width. 

The proclined maxillary and mandibular incisors were similar from T_1_ period to T_2_ period in both groups, which is consistent with the findings reported in other studies.^6^ ^,^ ^26^ ^,^ ^27^ In this present study, the cephalometric evaluations showed that the relief of the maxillary and mandibular anterior crowding mainly occurred as a result of labial inclination, independent of the type of bracket. Nevertheless, the studies in the literature^6^ ^,^ ^26^ ^,^ ^27^ did not evaluate long-term incisor position changes with self-ligating brackets. The results of the present study showed that the changes in the maxillary and mandibular dental measurements during the three-year follow-up period were insignificant in the conventional and self-ligating bracket groups. Similar to the present study, only Basciftci et al^15^ evaluated the long-term dentoalveolar effects of self-ligating brackets. However, these authors^15^ did not compare the experimental group to a control group. In keeping with the findings of the present study, the maxillary and mandibular incisor position changes were not significant from the immediate post-treatment period to the two-year follow-up period.^15^ However, different from the present study, they^15^ reported that the upper inter-canine width remained stable in the self-ligating bracket patients during all the retention periods. In the present study, some significant relapse was observed in the inter-canine width in both the self-ligating bracket group (1.18 mm) and the conventional bracket group (0.86 mm). This difference may be due to the retention protocol we used, since we did not apply upper and lower lingual retainers as Basciftci et al^15^ did in their study.

In the present study, the postero-anterior cephalometric evaluation measurements indicated significant buccal tipping of the upper molars in both treatment groups at the end of the treatment. In accordance with this finding, Yu et al.^28^ showed buccal tipping of the molars when using rapid palatal expansion (RPE) and the Damon technique with a non-extraction treatment approach. Cattaneo et al.^29^ also indicated buccal tipping in the self-ligating bracket group. It is known that increased tipping of the maxillary molars would put a patient at risk of future relapse. Therefore, different from other studies^14,15^ that investigated the stability of self-ligating brackets, the present study also evaluated the long-term effects on the upper molar inclination changes. A significant decrease in all frontal measurements was observed in both groups, indicating a significant relapse. The relapse amounts were not statistically different from each other when both groups were compared. However, the *p* value (0.055) of UL6-ML degrees (Table 3), which indicated a more clinically-significant relapse of the upper left molar teeth inclination in the Damon bracket group, must be taken into consideration. This result is probably due to the greater buccal tipping of the molars at the end of the treatment in the Damon bracket group, as found in a previous study.^9^ In the Damon bracket group, the low buccal root torque and the increased tipping of the maxillary molars could be considered as relapse risk factors. 

The limitation of this study might be its retrospective design. However, to reduce the disadvantage of potential selection bias,^30^ the same treatment protocol (the non-extraction treatment included the same archwire sequence with no other appliances) and the same retention protocol were applied by only one practitioner in the same clinic, while creating the samples. 

## CONCLUSION

In this study, no significant differences were found in terms of long-term stability between the self-ligating (Damon brackets) and the conventional (quad-helix appliance with conventional brackets) treatment systems. However, further long-term follow-up, randomized controlled trials are needed to precisely know how using self-ligating brackets impacts stability.
